# Association between being breastfed in infancy and adult colorectal cancer risk among Japanese men and women

**DOI:** 10.1038/s41598-024-60448-1

**Published:** 2024-04-26

**Authors:** Yuko Minami, Seiki Kanemura, Jun Kusaka, Makoto Kinouchi, Shinichi Suzuki, Hajime Iwasashi, Yoshikazu Nishino, Yoichiro Kakugawa, Koh Miura

**Affiliations:** 1 Department of Health Sciences, Tohoku University Graduate School of Medicine, 2-1 Seiryo-Machi, Aoba-Ku, Sendai, Miyagi 980-8575 Japan; 2 Division of Cancer Epidemiology and Prevention, Miyagi Cancer Center Research Institute, 47-1 Nodayama, Medeshima-Shiode, Natori, Miyagi 981-1293 Japan; 3 Center for Preventive Medicine, Osaki Citizen Hospital, 2-3-15 Senjuji-Machi, Furukawa, Osaki, Miyagi 989-6174 Japan; 4 Department of Gastroenterology, Miyagi Cancer Center Hospital, 47-1 Nodayama, Medeshima-Shiode, Natori, Miyagi 981-1293 Japan; 5 Department of Surgery, Miyagi Cancer Center Hospital, 47-1 Nodayama, Medeshima-Shiode, Natori, Miyagi 981-1293 Japan; 6 Deapartment of Epidemiology and Public Health, Kanazawa Medical University, 1-1 Daigaku, Uchinada, Kahoku, Ishikawa 920-0293 Japan; 7 Department of Surgery, Sendai Red Cross Hospital, 2-43-3 Honcho, Yagiyama, Sendai, Miyagi 982-8501 Japan; 8 Department of Surgery, Omagari Kosei Medical Center, 8-65 Omagari-Toricho, Daisen, Akita 014-0027 Japan

**Keywords:** Gastroenterology, Medical research, Oncology, Risk factors

## Abstract

It has been postulated that being breastfed in infancy affects not only health status in childhood but also disease risk in adulthood. To investigate the association of being breastfed with the risks of adult colorectal cancer and benign tumor, we conducted a case–control study including 1190 colorectal cancer and 1585 benign tumor cases and 5301 controls, admitted to a single hospital in Miyagi Prefecture, Japan, between 1997 and 2013. History of having been breastfed was assessed using a self-administered questionnaire, and odds ratios (ORs) were estimated using unconditional logistic regression. There was no association between being breastfed and colorectal cancer risk (breastfed versus formula-only fed, OR = 1.21; 95% CI 0.87–1.67). There was also no association with the risk of benign tumor (OR = 1.04). On the other hand, analyses stratified by sex and birth year found heterogeneous associations. Women born after 1950 who had been breastfed tended to have increased risks of colorectal cancer (OR = 1.58) and benign tumor (OR = 1.51) relative to those who had been formula-only fed, although not statistically significant. In men born after 1950, being breastfed was associated with a significantly decreased risk of benign tumor (OR = 0.57; 95% CI 0.33–0.98).

## Introduction

During the past several decades, the incidence of colorectal cancer has been increasing in Japan^1^. According to the latest report from the Japan National Cancer Registry, colorectal cancer is now the leading cancer in both sexes combined^2^. On the other hand, in Western countries, the incidence of younger-onset colorectal cancer has been increasing rapidly, despite the fact that the overall incidence of colorectal cancer has been declining or stabilizing^3,4^. These trends in overall incidence may be related to be changes in the prevalence of risk factors in individual countries, most of which pertain to lifestyles in adulthood such as obesity and alcohol drinking^5,6^. However, the recent increase in younger-onset colorectal cancer incidence in Western countries suggests that early life factors may affect colorectal cancer risk^7–9^. In Japan, some early life factors might also have been responsible for the increased overall incidence, in view of the drastic changes in lifestyle since the end of World War II^10^.

A number of studies have investigated the association between lifestyle during early life and colorectal cancer risk^11–14^. The US large cohort study has reported that obesity in early life might be associated with an increased risk of colorectal cancer in later life^13^. Other studies have indicated that individuals who have had severe energy restriction in adolescence are at lower risk^11,12^. Data suggest that dietary habits and nutrient intake during early life may affect subsequent colorectal cancer risk^9,14^. However, evidence related to early life risk factors has been limited and less consistent.

Breastfeeding in infancy, one such early life factor, may influence disease risk into adulthood. It has long been recognized that breastfed infants acquire health benefits such as lower risks of infection and obesity during childhood^15,16^. Such benefits may persist^17^, and may have a significant impact on the subsequent risk of adult diseases, including colorectal cancer. Furthermore, breast milk not only has nutritional benefits, but may also support the establishment of gut microbiota^16,18,19^. Recent studies have suggested that gut microbiome composition may be related to the risk of colorectal cancer^20–22^. Gut microbial differences between breastfed and formula-fed infants might have a meaningful impact on future disease risk^23^. To our knowledge, however, only four studies^24–27^ have investigated the association between breastfeeding in infancy and colorectal cancer risk, and their results were inconsistent. Two study found a significantly increased risk of colorectal cancer in women who had been breastfed^25,27^, whereas other two studies found no such association^24,26^.

Against this background, the present case–control study was conducted to investigate whether having been breastfed in infancy was associated with colorectal cancer risk in adulthood. Data on lifestyle and personal histories were collected from patients aged 30 years and over who had been admitted to a single hospital in Miyagi Prefecture, Japan, between 1997 and 2013. The patients were asked to report feeding practice (whether they had been breastfed or formula-fed) in infancy. Colorectal cancer risk was evaluated according to feeding practice. The relationship between having been breastfed and the risk of benign colorectal tumor, a precursor of colorectal cancer, was also investigated.

## Methods

### Study subjects and data collection

In January 1997, we began a questionnaire survey in connection with the present study. Information on lifestyle and personal histories, including history of having been breastfed, was collected from patients at their first admission to Miyagi Cancer Center Hospital (MCCH) using a self-administered questionnaire. The MCCH is located in the southern part of Miyagi Prefecture and functions as a hospital for both cancer and benign diseases. The data collection procedure has already been described elsewhere^28,29^. Briefly, on the day of reservation for the admission (i.e. 10–15 days before admission), nurses informed the patients of the purpose of the study and handed the questionnaires. The purpose of the study was also stated on the cover page of the questionnaire. On the actual day of admission, the nurses collected the signed questionnaires from the patients who consented to participate. At the MCCH, in principle, detailed diagnostic tests and initial treatment are initiated after admission. Therefore, data collected by the questionnaire survey were considered to be pretreatment or prediagnosis data. Between January 1997 and December 2013, the questionnaire was handed to 30,182 first-admitted patients, of whom 26,985 consented and responded (89.4%). Cases and controls were selected from among the respondents to the questionnaire survey.

To identify incident cases of colorectal cancer, a list of respondents for each survey year was linked to the MCCH hospital-based cancer registry database for that year. Through this linkage from 1997 to 2013, 26,985 respondents were classified into 2219 with history of cancer, 1576 with newly diagnosed colorectal cancer, 14,345 with newly diagnosed other cancers, and 8845 non-cancer patients. Of 1576 colorectal cancer patients, six patients under 30 years old were excluded, leaving 1570 patients. Among them, 113 patients with concurrent malignant tumors other than colorectal cancer were further excluded. Finally, 1457 were identified as incident cancer cases (1383 histologically confirmed colorectal cancer, 7 non-epithelial malignant tumor, and 67 malignancies without pathological data). We included patients with non-epithelial malignant tumors and those without pathological data in the cancer case group. The subsites in cancer cases were colon in 870, rectum in 564, and concurrent colon and rectum in 23.

Cases with benign tumor were identified using both the hospital-based cancer registry and the disease registration databases recording diagnostic information for all patients admitted to the MCCH. A list of respondents for each survey year was linked to these two databases for that year and a total of 1910 first-admitted patients aged 30 years and over without a history of cancer were selected as benign colorectal tumor cases. Subsite and pathological data were unavailable for these benign cases because the disease registration database did not cover this information.

Controls were selected from among the non-cancer respondents. Their diagnoses were confirmed by linking to the above two databases for each survey year. Among 8845 non-cancer patients, 568 under 30 years old were excluded. Accordingly, 8277 non-cancer patients aged 30 years and over without a history of cancer were selected as possible controls. After excluding 1910 patients with benign colorectal tumors from the possible controls, a total of 6367 non-cancer patients were identified as controls. In the present study, we included patients with benign tumors other than benign colorectal tumor in the control group. In hospital-based case–control studies, it is reasonable to exclude patients with diseases believed to be related to the exposure from the control group^30^. However, it is unclear whether exposure of the present study, namely being breastfed, is associated with the risk of benign tumors. Among the controls, the diagnoses were cardiovascular disease in 224, respiratory tract disease in 324, digestive tract disease in 974, benign tumor in 2932, other benign diseases in 1037, and no abnormal findings in 876. The sites of the benign tumors were the stomach in 343, lung in 51, breast in 49, gynecologic organs in 426, bone or connective tissue in 1298, and others in 765.

This study was approved by the ethical review board of the Miyagi Cancer Center (Protocol Identification Number 2021–063) and was conducted in accordance with the principles specified in the Declaration of Helsinki.

### Assessment of exposures

In order to assess individual history of having been breastfed in infancy, patients were asked: “By which of the following practices were you fed as a baby?”. Response categories were: breast only, mixed breast and formula, formula only, don’t know. Of the study subjects, 379 patients who did not respond to this question (60 cancer cases, 60 benign tumor cases, 259 controls) were excluded from the subsequent analysis. Furthermore, 1279 subjects who chose “don’t know” (207 cancer cases, 265 benign tumor cases, 807 controls) were also excluded, leaving a total of 8076 patients as the analyzed subjects (1190 cancer cases, 1585 benign tumor cases, 5301 controls).

Two sorts of exposure were considered in this study. One consisted of 3 categories, excluding the “don’t know” category (i.e. breast only, mixed breast and formula, formula only). For the other exposure, the two categories “breast only” and “mixed breast and formula” were combined into a single category (“breastfed” group).

### Statistical analysis

We used unconditional logistic regression analysis to estimate odds ratios (ORs) and 95% confidence intervals (CIs) for the risks of colorectal cancer and benign tumor in relation to the above two sorts of exposures. Taking “formula only” as a reference category, the OR for each category was estimated. Analyses were performed according to sex as well as for both sexes combined. Analyses stratified by birth year (< 1950, ≥ 1950) were also performed. In Japan, commercial formula milk was first introduced in 1917 and the standard formula composition was proposed in 1950^31,32^. The quality of formula has improved since 1950. For colorectal cancer, analysis stratified by subsite (colon and rectum) was also conducted. To assess risk heterogeneity across sex, we conducted Wald tests with an interaction term between sex and exposure.

We considered the following variables to be potential confounders: age, year of survey, area of residence (area surrounding the hospital, i.e., southern Miyagi Prefecture, other area), referral status (from screening, other), occupation (professional or office work, other, missing), alcohol drinking (never, ever), smoking (never, ever), body mass index (BMI) (< 18.5, ≥ 18.5– < 25.0, ≥ 25.0), time spent exercising (almost no, ≥ 1 h per week), intakes of processed meat, milk and seaweed (never or few times per month, 1–2 times per week, 3–4 times per week, every day), and family history of colorectal cancer (absent, present), some of which have been regarded as convincing or probable risk factors for colorectal cancer^5,6^. The information regarding referral status was obtained from all study subjects using the questionnaire. “Screening” includes any screening. Although the association between intake of seaweed, food containing dietary fiber, and colorectal cancer risk has not yet been established^5,33^, our previous study indicated that seaweed intake might be associated with improved survival in patients with colorectal cancer^34^. Missing values for confounders were treated as an additional variable category. Furthermore, Birthplace was also controlled for. The birthplace response categories in the questionnaire were urban, rural, mountainous, seaside, and other. For the analysis, we regrouped these five categories into two classes (urban, rural or other). Some previous reports in Japan have suggested that urban living conditions may have been favorable for infant nutritional status, at least among subjects born after 1950^35,36^.

The results were regarded as statistically significant if the two-sided *P* values were < 0.05. All statistical analyses were performed using SAS software (version 9.4; SAS Institute, Cary, NC).

## Results

### Characteristics of study subjects

Table 1 shows the characteristics of the study subjects, i.e., the distribution of confounders among cases and controls. A relatively large number of benign tumors were detected by screening. For female subjects, the distributions of smoking and drinking status were similar between cases (cancer, benign tumor) and controls. On the other hand, male cases with benign tumor tended to be drinkers in comparison with controls. In both sexes, benign tumor cases tended to be obese. With regard to food intake, the distributions of processed meat, milk and seaweed intake were comparable between cases (cancer, benign tumor) and controls. The frequency of subjects with a family history of colorectal cancer was higher among the cases (cancer, benign tumor) relative to the controls.

**Table 1:** Characteristics of colorectal cancer and benign tumor cases and controls by sex.

Factor		Cases				Controls	
		Cancer		Benign tumor			
		M^a^	W^b^	M	W	M	W
Number of subjects		668	522	1045	540	2438	2863
Age group (years old) (%)	30–39	1.5	1.5	1.7	1.7	6.2	10.7
	40–49	7.5	6.3	7.8	9.6	12.8	18.1
	50–59	18.6	19.0	24.9	16.9	20.3	22.0
	60–69	36.2	27.4	35.9	37.0	27.9	23.3
	70–79	28.4	31.2	24.1	27.4	26.0	19.5
	80-	7.8	14.6	5.5	7.4	6.8	6.3
	Mean (years old)	65.1	66.6	63.5	64.7	62.0	58.6
	SD	10.8	11.7	10.6	11.0	13.1	14.2
Year of survey (%)	1997–2005	58.2	54.2	54.1	49.6	67.6	65.9
	2006–2013	41.8	45.8	45.9	50.4	32.4	34.1
Area of residence (%)	Southern Miyagi prefecture	86.5	87.7	95.1	94.8	86.1	87.3
	Other area	13.5	12.3	4.9	5.2	13.9	12.7
Referral status (%)	From screening	23.6	20.5	37.5	46.7	11.3	14.2
	Other	76.4	79.5	62.5	53.3	88.7	85.8
Occupation (%)	Professional or office work	30.1	19.9	35.1	19.8	31.2	24.7
	Industrial work or fishery	44.0	23.6	43.5	21.9	44.1	26.0
	Agriculture or forestry	14.4	11.3	9.8	13.9	14.4	9.1
	Other^c^	2.1	21.5	2.1	28.0	2.0	24.4
	Missing	9.4	23.7	9.5	16.4	8.3	15.8
Birthplace (%)	Urban	15.0	18.2	15.1	18.7	17.3	17.1
	Rural or other	83.7	79.9	84.2	79.3	82.0	80.4
	Missing	1.3	1.9	0.7	2.0	0.7	2.5
Alcohol drinking (%)	Ever	78.0	18.6	82.9	22.0	73.3	24.5
	Never	20.1	75.5	15.5	73.2	25.2	71.1
	Missing	1.9	5.9	1.6	4.8	1.5	4.4
Smoking (%)	Ever	75.2	13.8	76.0	11.9	75.4	17.0
	Never	23.2	81.4	23.0	83.1	23.2	79.5
	Missing	1.6	4.8	1.0	5.0	1.4	3.5
BMI (kg/m^2^, %)	< 18.5	5.1	8.4	3.0	5.2	5.1	6.9
	18.5 ≤ < 25.0	66.6	65.3	66.5	60.2	65.8	62.7
	25.0 ≤	28.0	26.1	30.3	31.7	28.3	27.5
	Missing	0.3	0.2	0.2	3.0	0.8	2.8
Time spent exercising (%)	Almost no	41.9	39.8	36.4	35.2	40.8	49.1
	≥ 1 h per week	52.5	50.8	57.9	57.4	52.3	44.4
	Missing	5.5	9.4	5.7	7.4	7.0	6.5
Seaweed intake (%)	Never or few times per month	13.5	8.6	11.8	7.0	10.8	7.8
	1–2 times per week	29.6	30.8	35.3	27.8	35.6	31.8
	3–4 times per week	33.4	35.1	32.0	40.0	32.0	36.0
	Everyday	17.2	21.1	16.2	21.1	16.7	20.4
	Missing	6.3	4.4	4.8	4.1	4.8	4.0
Processed meat intake (%)	Never or few times per month	47.3	48.3	48.1	44.4	45.3	42.8
	1–2 times per week	28.1	29.9	31.4	33.7	30.3	36.2
	3–4 times per week	4.9	4.4	6.5	7.8	7.5	8.0
	Everyday	1.9	1.5	1.2	0.7	1.4	1.4
	Missing	17.7	15.9	12.7	13.3	15.5	11.6
Cow’s milk intake (%)	Never or few times per month	26.9	25.1	28.3	23.7	24.9	22.0
	1–2 times per week	16.0	17.8	16.3	12.6	15.1	15.0
	3–4 times per week	14.2	11.3	13.7	16.5	13.8	15.2
	Everyday	33.7	37.2	34.3	38.9	38.5	42.5
	Missing	9.1	8.6	7.4	8.3	7.7	5.3
Family history of colorectal cancer in first-degree relatives^d^ (%)	Absent	88.6	85.8	87.0	89.3	93.7	93.8
	Present	11.4	14.2	13.0	10.7	6.3	6.2

^a^M, Men.

^b^W, Women.

^c^Household wife/Domestic help/Other.

^d^History in parents and siblings.

The distribution of exposure (feeding practice) is shown in Table 2. According to birth year, the frequency of subjects that had been formula-fed tended to be high among subjects born after 1950. Among subjects born before 1940, over 90% of subjects had been fed by breast only.

**Table 2:** Feeding practice in infancy according to birth year.

Birth year	No. and % of cancer cases				No. and % of benign tumor cases				No. and % of controls			
	Breast only	Mixed breast and formula	Formula only	Total	Breast only	Mixed breast and formula	Formula only	Total	Breast only	Mixed breast and formula	Formula only	Total
–1919	43 (97.7)	1 (2.3)	0 (0.0)	44 (100)	29 (87.9)	3 (9.1)	1 (3.0)	33 (100)	151 (93.8)	9 (5.6)	1 (0.6)	161 (100)
1920–1929	253 (95.8)	9 (3.4)	2 (0.8)	264 (100)	207 (92.0)	12 (5.3)	6 (2.7)	225 (100)	755 (91.8)	37 (4.5)	30 (3.6)	822 (100)
1930–1939	328 (90.9)	20 (5.5)	13 (3.6)	361 (100)	444 (92.9)	24 (5.0)	10 (2.1)	478 (100)	1223 (91.9)	72 (5.4)	35 (2.6)	1330 (100)
1940–1949	256 (88.0)	24 (8.2)	11 (3.8)	291 (100)	450 (87.9)	39 (7.6)	23 (4.5)	512 (100)	1065 (87.8)	100 (8.3)	47 (3.9)	1212 (100)
1950–1959	132 (78.6)	23 (13.7)	13 (7.7)	168 (100)	208 (83.5)	24 (9.6)	17 (6.8)	249 (100)	771 (76.3)	165 (16.3)	74 (7.3)	1010 (100)
1960–1969	24 (52.2)	17 (36.9)	5 (10.9)	46 (100)	29 (40.8)	25 (35.2)	17 (23.9)	71 (100)	244 (45.9)	197 (37.1)	90 (17.0)	531 (100)
1970–	8 (50.0)	5 (31.2)	3 (18.8)	16 (100)	3 (17.6)	12 (70.6)	2 (11.8)	17 (100)	70 (29.8)	89 (37.9)	76 (32.3)	235 (100)
Total	1044 (87.7)	99 (8.3)	47 (4.0)	1190 (100)	1370 (86.4)	139 (8.8)	76 (4.8)	1585 (100)	4279 (80.7)	669 (12.6)	353 (6.7)	5301 (100)

% in parenthesis.

### Being breastfed in infancy and the risks of colorectal cancer and benign tumor in both sexes combined

Table 3 shows the ORs and 95% CI for feeding practice according to colorectal cancer and benign tumor, based on combined analysis for both sexes. Analysis adjusted for age, sex and year of survey showed that having been breastfed was not associated with risk for either colorectal cancer or benign tumor. Further adjustment for confounding variables (multivariate-adjusted analysis) did not change the value of OR (OR of cancer = 1.21; 95% CI 0.87–1.67, OR of benign tumor = 1.04; 95% CI 0.78–1.38). There was also no evident association for either mixed breast and formula or breast only. Analysis according to birth year showed no significant association for either cancer or benign tumor.

**Table 3:** Association between breastfed in infancy and the risk of colorectal tumors according to cancer and benign tumor in both sexes combined.

Group	Category	Cancer				Benign tumor			
		Cases (n)	Controls (n)	Age, sex and year of survey-adjusted	Multivariate-adjusted^b^	Cases (n)	Controls (n)	Age, sex and year of survey-adjusted	Multivariate-adjusted^b^
				OR (95% CI)	OR (95% CI)			OR (95% CI)	OR (95% CI)
All	Formula only	47	353	1.00 (Ref)	1.00 (Ref)	76	353	1.00 (Ref)	1.00 (Ref)
	Breastfed^a^	1143	4948	1.22 (0.89–1.69)	1.21 (0.87–1.67)	1509	4948	1.08 (0.83–1.41)	1.04 (0.78–1.38)
	Mixed breast and formula	99	669	1.06 (0.73–1.54)	1.07 (0.73–1.56)	139	669	0.89 (0.65–1.22)	0.84 (0.60–1.18)
	Breast only	1044	4279	1.26 (0.91–1.74)	1.24 (0.89–1.72)	1370	4279	1.13 (0.87–1.48)	1.09 (0.82–1.45)
Birth year < 1950	Formula only	26	113	1.00 (Ref)	1.00 (Ref)	40	113	1.00 (Ref)	1.00 (Ref)
	Breastfed	934	3412	1.12 (0.73–1.73)	1.15 (0.74–1.78)	1208	3412	0.94 (0.65–1.37)	1.02 (0.68–1.52)
	Mixed breast and formula	54	218	1.07 (0.64–1.81)	1.11 (0.65–1.89)	78	218	0.98 (0.63–1.54)	1.00 (0.62–1.63)
	Breast only	880	3194	1.13 (0.73–1.74)	1.15 (0.74–1.79)	1130	3194	0.94 (0.65–1.37)	1.02 (0.68–1.52)
Birth year ≥ 1950	Formula only	21	240	1.00 (Ref)	1.00 (Ref)	36	240	1.00 (Ref)	1.00 (Ref)
	Breastfed	209	1536	1.09 (0.66–1.78)	1.15 (0.69–1.92)	301	1536	0.82 (0.55–1.23)	0.80 (0.52–1.24)
	Mixed breast and formula	45	451	0.98 (0.56–1.71)	1.02 (0.57–1.83)	61	451	0.72 (0.46–1.15)	0.67 (0.41–1.11)
	Breast only	164	1085	1.14 (0.69–1.89)	1.21 (0.72–2.05)	240	1085	0.87 (0.57–1.32)	0.88 (0.56–1.38)

^a^Mixed breast and formula or breast only.

^b^Adjusted for age, sex, year of survey, area of residence (southern Miyagi, other area), referral status (from screening, other), occupation (professional or office work, other, missing), birth place (urban, rural or other, missing), alcohol drinking (never, ever, missing), smoking (never, ever, missing), BMI (< 18.5, ≥ 18.5– < 25.0, ≥ 25.0, missing), time spent exercising (almost no, ≥ 1 h per week, missing), seaweed intake (never or few times per month, 1–2 times per week, 3–4 times per week, everyday, missing), processed meat intake (never or few times per month, 1–2 times per week, 3–4 times per week, everyday, missing), cow’s milk intake (never or few times per month, 1–2 times per week, 3–4 times per week, everyday, missing), family history of colorectal cancer (absent, present).

### Being breastfed in infancy and the risk of colorectal cancer according to sex

Table 4 shows colorectal cancer risk in relation to feeding practice according to sex. Overall analysis revealed no significant association with feeding practice for either men or women, although having been breastfed tended to be positively associated with colorectal cancer risk in women (OR = 1.48; 95% CI 0.91–2.39). However, analysis by birth year indicated a different magnitude of OR across birth year and sex. Among subjects born before 1950, the ORs for mixed breast and formula, and breast only were around one in either men or women. On the other hand, the direction of risk for breastfed among subjects born after 1950 differed between men and women: there was an inverse association in men (OR = 0.73; 95% CI 0.36–1.47) and a positive association in women (OR = 1.58; 95% CI 0.72–3.49), although neither risk was statistically significant. According to mixed breast and formula, and breast only, a relatively large OR was observed for women who had been fed by breast only, although the value did not reach statistical significance (OR = 1.84; 95% CI 0.82–4.13).

**Table 4:** Association between breastfed in infancy and the risk of colorectal cancer according to sex.

Group	Category	Men				Women				Heterogeneity across sex^c^
		Cases (n)	Controls (n)	Age and year of survey-adjusted	Multivariate-adjusted^b^	Cases (n)	Controls (n)	Age and year of survey-adjusted	Multivariate-adjusted^b^	
				OR (95% CI)	OR (95% CI)			OR (95% CI)	OR (95% CI)	
All	Formula only	26	115	1.00 (Ref)	1.00 (Ref)	21	238	1.00 (Ref)	1.00 (Ref)	0.07
	Breastfed^a^	642	2323	1.03 (0.66–1.61)	0.93 (0.59–1.47)	501	2625	1.39 (0.86–2.22)	1.48 (0.91–2.39)	
	Mixed breast and formula	56	255	0.95 (0.57–1.60)	0.90 (0.53–1.53)	43	414	1.10 (0.63–1.91)	1.15 (0.65–2.02)	
	Breast only	586	2068	1.05 (0.67–1.63)	0.94 (0.60–1.48)	458	2211	1.45 (0.90–2.33)	1.55 (0.96–2.52)	
Birth year < 1950	Formula only	14	50	1.00 (Ref)	1.00 (Ref)	12	63	1.00 (Ref)	1.00 (Ref)	0.58
	Breastfed	526	1711	1.07 (0.58–1.95)	1.01 (0.54–1.86)	408	1701	1.18 (0.63–2.23)	1.33 (0.69–2.53)	
	Mixed breast and formula	27	86	1.08 (0.52–2.26)	1.08 (0.51–2.28)	27	132	1.08 (0.51–2.29)	1.17 (0.54–2.52)	
	Breast only	499	1625	1.07 (0.58–1.95)	1.00 (0.54–1.85)	381	1569	1.19 (0.63–2.25)	1.34 (0.70–2.56)	
Birth year ≥ 1950	Formula only	12	65	1.00 (Ref)	1.00 (Ref)	9	175	1.00 (Ref)	1.00 (Ref)	0.08
	Breastfed	116	612	0.81 (0.41–1.59)	0.73 (0.36–1.47)	93	924	1.43 (0.69–2.99)	1.58 (0.72–3.49)	
	Mixed breast and formula	29	169	0.85 (0.40–1.82)	0.74 (0.33–1.62)	16	282	1.05 (0.45–2.47)	1.06 (0.42–2.68)	
	Breast only	87	443	0.79 (0.39–1.57)	0.72 (0.35–1.49)	77	642	1.64 (0.77–3.48)	1.84 (0.82–4.13)	

^a^Mixed breast and formula or breast only.

^b^Adjusted for age, year of survey, area of residence (southern Miyagi, other area), referral status (from screening, other), occupation (professional or office work, other, missing), birth place (urban, rural or other, missing), alcohol drinking (never, ever, missing), smoking (never, ever, missing), BMI (< 18.5, ≥ 18.5– < 25.0, ≥ 25.0, missing), time spent exercising (almost no, ≥ 1 h per week, missing), seaweed intake (never or few times per month, 1–2 times per week, 3–4 times per week, everyday, missing), processed meat intake (never or few times per month, 1–2 times per week, 3–4 times per week, everyday, missing), cow’s milk intake (never or few times per month, 1–2 times per week, 3–4 times per week, everyday, missing), family history of colorectal cancer (absent, present).

^c^*p* for interaction between sex*breastfed and formula only-fed in multivariate analysis.

### Being breastfed in infancy and the risk of benign colorectal tumor according to sex

Table 5 shows the risk of developing benign tumor by sex. Overall association with feeding practice was unity in both men and women. However, analyses according to birth year indicated that risk profiles might differ between men and women in each birth year group. Among subjects who were born after 1950, having been breastfed tended to show a positive association in women (OR = 1.51; 95% CI 0.66–3.45), reflecting a direction similar to that for cancer risk shown in Table 4. On the other hand, a significant inverse association between having been breastfed and benign tumor risk was found in men (OR = 0.57; 95% CI 0.33–0.98). The direction of this risk in men was also similar to that for cancer risk shown in Table 4. Heterogeneity test for the OR between men and women gave a significant result (*p* = 0.03). Analysis among subjects born before 1950 yielded a positive association with breastfed in men (OR = 1.67; 95% CI 0.89–3.14). This association was different from that in women (heterogeneity *p* = 0.04).

**Table 5:** Association between breastfed in infancy and the risk of benign colorectal tumor according to sex.

Group	Category	Men				Women				Heterogeneity across sex^c^
		Cases (n)	Controls (n)	Age and year of survey-adjusted	Multivariate-adjusted^b^	Cases (n)	Controls (n)	Age and year of survey-adjusted	Multivariate-adjusted^b^	
				OR (95% CI)	OR (95% CI)			OR (95% CI)	OR (95% CI)	
All	Formula only	43	115	1.00 (Ref)	1.00 (Ref)	33	238	1.00 (Ref)	1.00 (Ref)	0.50
	Breastfed^a^	1002	2323	1.08 (0.75–1.55)	1.06 (0.72–1.58)	507	2625	1.05 (0.71–1.55)	0.99 (0.65–1.51)	
	Mixed breast and formula	88	255	0.87 (0.57–1.34)	0.86 (0.54–1.36)	51	414	0.88 (0.54–1.41)	0.79 (0.48–1.31)	
	Breast only	914	2068	1.13 (0.78–1.63)	1.11 (0.75–1.66)	456	2211	1.09 (0.73–1.62)	1.04 (0.68–1.59)	
Birth year < 1950	Formula only	16	50	1.00 (Ref)	1.00 (Ref)	24	63	1.00 (Ref)	1.00 (Ref)	0.04
	Breastfed	794	1711	1.46 (0.83–2.60)	1.67 (0.89–3.14)	414	1701	0.65 (0.40–1.06)	0.68 (0.40–1.15)	
	Mixed breast and formula	47	86	1.62 (0.83–3.16)	1.85 (0.89–3.86)	31	132	0.64 (0.34–1.19)	0.57 (0.29–1.10)	
	Breast only	747	1625	1.46 (0.82–2.58)	1.66 (0.89–3.12)	383	1569	0.65 (0.40–1.06)	0.69 (0.41–1.17)	
Birth year ≥ 1950	Formula only	27	65	1.00 (Ref)	1.00 (Ref)	9	175	1.00 (Ref)	1.00 (Ref)	0.03
	Breastfed	208	612	0.57 (0.34–0.95)	0.57 (0.33–0.98)	93	924	1.52 (0.73–3.16)	1.51 (0.66–3.45)	
	Mixed breast and formula	41	169	0.51 (0.29–0.92)	0.47 (0.25–0.88)	20	282	1.32 (0.58–2.99)	1.25 (0.50–3.13)	
	Breast only	167	443	0.60 (0.36–1.02)	0.62 (0.35–1.09)	73	642	1.65 (0.77–3.50)	1.69 (0.72–3.95)	

^a^Mixed breast and formula or breast only.

^b^Adjusted for age, year of survey, area of residence (southern Miyagi, other area), referral status (from screening, other), occupation (professional or office work, other, missing), birth place (urban, rural or other, missing), alcohol drinking (never, ever, missing), smoking (never, ever, missing), BMI (< 18.5, ≥ 18.5– < 25.0, ≥ 25.0, missing), time spent exercising (almost no, ≥ 1 h per week, missing), seaweed intake (never or few times per month, 1–2 times per week, 3–4 times per week, everyday, missing), processed meat intake (never or few times per month, 1–2 times per week, 3–4 times per week, everyday, missing), cow’s milk intake (never or few times per month, 1–2 times per week, 3–4 times per week, everyday, missing), family history of colorectal cancer (absent, present).

^c^*p* for interaction between sex*breastfed and formula only-fed in multivariate analysis.

### Being breastfed in infancy and the risk of colorectal cancer according to subsite

Table 6 shows the association between feeding practice and colorectal cancer risk according to subsite. In both sex combined analysis, having been breastfed was not associated with the risk of either colon or rectal cancer, although the direction of risk tended to be different between colon and rectum. Further stratification by sex also showed no significant association. However, men who had been breastfed tended to have reduced risk of rectal cancer (OR = 0.69; 95% CI 0.39–1.20). The interaction term for assessment of risk heterogeneity across sex was marginally significant in rectal cancer (*p* = 0.06). Analysis by sex and birth year was not performed due to the limited number of cases and controls.

**Table 6:** Association between breastfed in infancy and the risk of colorectal cancer according to subsite.

Group	Category	Controls (n)	Colon		Rectum	
			Cases (n)	OR (95% CI)^b^	Cases (n)	OR (95% CI)
All	Formula only	353	22	1.00 (Ref)	25	1.00 (Ref)
	Breastfed^a^	4948	690	1.48 (0.94–2.34)	434	0.96 (0.62–1.48)
	Mixed breast and formula	669	60	1.42 (0.85–2.39)	39	0.78 (0.46–1.32)
	Breast only	4279	630	1.50 (0.95–2.36)	395	1.01 (0.65–1.56)
Men	Formula only	115	10	1.00 (Ref)	16	1.00 (Ref)
	Breastfed	2323	361	1.38 (0.70–2.74)	268	0.69 (0.39–1.20)
	Mixed breast and formula	255	29	1.41 (0.65–3.06)	27	0.64 (0.33–1.26)
	Breast only	2068	332	1.38 (0.69–2.74)	241	0.70 (0.40–1.23)
Women	Formula only	238	12	1.00 (Ref)	9	1.00 (Ref)
	Breastfed	2625	329	1.60 (0.86–2.97)	166	1.36 (0.66–2.80)
	Mixed breast and formula	414	31	1.44 (0.71–2.93)	12	0.86 (0.35–2.11)
	Breast only	2211	298	1.63 (0.88–3.04)	154	1.49 (0.72–3.09)
Heterogeneity across sex^c^			0.61		0.06	

^a^Mixed breast and formula or breast only.

^b^Adjusted for age, sex, year of survey, area of residence (southern Miyagi, other area), referral status (from screening, other), occupation (professional or office work, other, missing), birth place (urban, rural or other, missing), alcohol drinking (never, ever, missing), smoking (never, ever, missing), BMI (< 18.5, ≥ 18.5– < 25.0, ≥ 25.0, missing), time spent exercising (almost no, ≥ 1 h per week, missing), seaweed intake (never or few times per month, 1–2 times per week, 3–4 times per week, everyday, missing), processed meat intake (never or few times per month, 1–2 times per week, 3–4 times per week, everyday, missing), cow’s milk intake (never or few times per month, 1–2 times per week, 3–4 times per week, everyday, missing), family history of colorectal cancer (absent, present).

^c^*p* for interaction between sex*breastfed and formula only-fed in multivariate analysis.

## Discussion

In this hospital-based case–control study, we evaluated the association of having been breastfed with the risk of colorectal cancer and benign tumor using self-reported information. There was no overall association between having been breastfed and colorectal cancer risk. No association was also observed for the risk of benign colorectal tumor. On the other hand, analyses stratified by sex and birth year revealed heterogeneous associations. Analysis of the subjects born before 1950 showed that having been breastfed was not associated with colorectal cancer risk in either men or women. Among subjects who had been born after 1950, having been breastfed tended to be positively associated with colorectal cancer risk in women and inversely associated in men, although neither risk was statistically significant. With regard to the risk of benign tumor, men born after 1950 had a significantly reduced risk if they had been breastfed. In general, epidemiological studies like the present one designed to investigate the long-term effects of early life factors, such as having been breastfed, are associated with difficulties because the data rely on recalled information on distant past life events^8,37^. Therefore, the results obtained from such studies must be interpreted with caution. However, being breastfed may be a potentially important early life factor associated with colorectal cancer risk, because breast milk is a diet-related exposure during the earliest period of life. This was our rationale for reporting the present results. To our knowledge, colorectal cancer risk associated with having been breastfed has never been investigated in a Japanese population.

It has been suggested that having been breastfed in infancy might affect not only health status in childhood but also disease risk in adulthood^15–17^. The recent UK biobank study showed that having been breastfed in infancy was associated with a lower risk of all-cause mortality in middle and late adulthood^26^. A lower risk was also observed for cardiovascular disease mortality and respiratory disease mortality, but there was no association with either cancer or colorectal cancer mortality. For colorectal cancer risk, another study using the Boyd Orr Cohort in UK has also reported no association with having been breastfed^24^. In the present study, feeding practice in infancy had no substantial effect on the risk of adult colorectal cancer, or the risk according to subsite (colon, rectum). The association of having been breastfed with the risk of benign colorectal tumor was also unity. These results were consistent with those of the above UK studies^24,26^. Although having been breastfed might have had a long-term impact on carcinogenesis through immune and metabolic responses^16,18^, its overall effect on colorectal cancer risk may be small and undetectable. It seems more likely that exposures to exogenous environments, including nutrient intake, during early- and mid-adulthood might have a larger impact on colorectal cancer risk. For example, migrant studies including ours have indicated that a late-stage promotional effect of the adult environment might play an important role in the development of colorectal cancer^38,39^. The recent epidemiological study based on the Nurses’ Health Study II showed that adherence to the WCRF/AICR cancer prevention recommendations during adulthood, but not during adolescence, was associated with a lower risk colorectal adenoma^40^.

Although an overall effect of having been breastfed on colorectal cancer risk was not detectable, as mentioned above, our analysis according to sex and birth year suggested that the effect of having been breastfed on colorectal cancer risk might differ between men and women. Women who had been breastfed, especially those born after 1950, tended to have increased risks of colorectal cancer and benign tumor, relative to those who had been formula- only fed. Men born after 1950 had a significantly reduced risk of benign tumor if they had been breastfed. Increased risks among women who had been breastfed were also observed in the prospective Million Women study in UK and the Nurses’ Health studies in US^25,27^. With regard to the risks associated with breast and formula feeding, these two studies provided the following hypothetical mechanisms^25,27^. First, long-term differences in gut microbiota between breastfed and formula-fed infants might affect colorectal cancer risk. Second, carcinogenic viruses might be transmitted vertically through breastmilk. Third, consumption of cow’s milk, the source of formula, might be linked to a decreased risk of colorectal cancer, although previous studies have yielded inconsistent results for the association between childhood dairy product intake and colorectal cancer risk^14,41^. Our results according to sex and birth year may be explained by these hypothetical mechanisms. Historically, in Japan, consumption of cow’s milk and dairy products has been low^42,43^. In comparison with Western countries, formulas including powdered cow’s milk were rarely used for infant nutrition until the end of World War II. Therefore, the results for women born after 1950 are considered to be comparable with those from the UK and US studies. On the other hand, it is unknown why colorectal cancer risk and benign tumor risk associated with breast milk and formula intakes might differ between men and women. To our knowledge, no previous studies have examined the risks in men. In terms of child growth, some studies have shown that exclusive breastfeeding may protect against obesity to a greater extent in boys than girls through microbiome development^44,45^. It has also been suggested that body fatness in early life might be associated with colorectal cancer risk^13^. Gut microbiome development during puberty may also be affected by infant characteristics including sex^46,47^. Although speculative, development of obesity during early life, possibly affecting future colorectal cancer risk, may be modified by such a sex-specific association between being breastfed and the gut microbiome. With regard to the role of cow’s milk as a source of formula, one study has shown that consumption of cow’s milk and dairy products during childhood may have differential effects on prepubertal growth and IGF-1 level between boys and girls, thus consequently modifying the risk of colorectal cancer^48^. However, evidence according to sex has been limited. Furthermore, the exogenous environment during adulthood may also have substantial effects on gut microbial composition and body fatness^49,50^. Thus, it remains unclear whether the risk associated with being breastfed is independent of the risk associated with the adult environment. To clarify the sex-specific associations between having been breastfed and colorectal cancer risk, further studies are required.

When assessing the association of having been breastfed with cancer risk, we may also need to consider the mother’s lifestyle factors and socioeconomic status (SES), as well as biological disease mechanisms in relation to infant feeding. Colorectal cancer risk associated with being breastfed may result not only from the biological effects of breastmilk, but also the perinatal environment. Maternal lifestyle factors and SES during the perinatal period, which influence lactation performance^43,45,51,52^, may have long-term effects on the child’s health and lifestyle^53^, and these effects may persist into adulthood. Although the role of the perinatal environment was discussed in our previous study focusing on breast cancer risk^29^, our more recent study revealed that the mother’s education level, occupation, lifestyle, and diet during pregnancy were related to exclusive breastfeeding, and that maternal nutrient intakes might have differential effects on breast milk production for boys and girls^52^. It is likely that maternal lifestyle factors and SES relating to breastfeeding may confound the risk associated with having been breastfed. In future studies, maternal factors should also be collected and considered.

The present study had both strengths and limitations. In hospital-based case–control studies such as the present one, some methodological limitations are likely to influence the results. First, we considered comparability between the cases and the controls, both of whom were selected from patients who had been admitted for the first time to a single hospital and had responded to a questionnaire survey. The survey response rate was high (89.4%). Diagnoses of the cases and controls were confirmed using the hospital-based cancer registry and the disease registration databases. However, different distributions between cases and controls made it difficult to match them individually based on factors such as age and year of survey. Alternatively, to improve comparability between the cases and controls, our statistical analyses appropriately controlled for background characteristics such as area of residence and referral status, as well as age and year of survey. Furthermore, additional analyses by dividing the data into two periods (1997–2005, 2006–2013) each showed a similar direction of risk as that for entire period (data not shown in tables). Thus, it is unlikely that problems of comparability between cases and controls would have distorted the results. On the other hand, the generalizability of our results may have been limited, because our study was performed at a single hospital in Japan. Second, the possibility of information bias must be considered. Since the questionnaire survey was performed at the time of first admission before any definite diagnosis or treatment, any information bias would have been generally minimal. However, self-reported information on exposure, i.e., having been breastfed in the distant past, might have been vulnerable to misclassification. To evaluate the reliability of this questionnaire item on feeding practice, subsamples of study subjects (n = 288) were re-surveyed about 1 year later using the same questionnaire. For the original four response categories including “don’t know”, the kappa statistic was moderate (κ = 0.43; 95% CI 0.31–0.53), indicating moderate reproducibility^54^. This kappa statistic was smaller than in our previous study^29^, possibly because the study subjects were older and had more difficulty in recalling their life histories. However, subjects who had been formula-fed would have increased following the introduction of standard formula in 1950 (Table 2). The kappa statistic calculated for subjects born after 1950 indicated substantial reproducibility (κ = 0.62; 95% CI 0.35–0.89 for original four categories). In addition, the distributions of feeding practice (Table 2) among the controls were similar to those in the contemporaneous maternal report^43^. It is unlikely that the distributions of exposure in the present study would have differed substantially from those in the general population. Furthermore, the hypothesis on which our study was based was not known to the study subjects. Therefore, any misclassification of exposure would have been non-differential^55^. Thus, information bias pertaining exposure is unlikely to have seriously distorted the results. To precisely evaluate the association between feeding practice in infancy and colorectal cancer risk, birth cohort studies are needed^37^. However, completion of such birth cohort studies requires a very long time period^24,41^. Therefore, at the present time, observational studies like the present one would seem to provide useful information.

Our study strengths were as follows. First, a relatively large number of cases and controls were included. Consequently, we were able to evaluate sex- and birth year-specific risk, as well as overall risk. Another strength was that established risk factors for colorectal cancer such as BMI, alcohol drinking, and processed meat intake, and birthplace were controlled for in the analysis, although residual confounding may have occurred. Birthplace may be a surrogate for the general environment during childhood.

In conclusion, this hospital-based case–control study has found no overall association between having been breastfed and colorectal cancer risk. There was also no evident association for the risk of benign colorectal tumor, which is a precursor of colorectal cancer. According to sex and birth year, women who had been breastfed, especially those born after 1950, tended to have increased risks of colorectal cancer and benign tumor, relative to those who had been formula only-fed, although not statistically significant. In men born after 1950, having been breastfed was associated with a significantly decreased risk of benign tumor. Although breastfeeding might have had a long-term impact on carcinogenesis through immune and metabolic responses, its overall effect on colorectal cancer risk may be small and undetectable. However, the differences in risk according to sex may indicate that biologic and environmental factors related to being breastfed, for example, gut microbiome composition mediated by breast milk and maternal lifestyle factors and SES during the perinatal period, might have differential impacts on the risks for adult colorectal cancer and benign tumor between men and women.

## Data Availability

The datasets generated and/or analyzed during the current study are not publicly available due to ethical restrictions in hospital-based research but are available from the corresponding author on reasonable request.
